# A physiological model for interpretation of arterial spin labeling reactive hyperemia of calf muscles

**DOI:** 10.1371/journal.pone.0183259

**Published:** 2017-08-24

**Authors:** Hou-Jen Chen, Graham A. Wright

**Affiliations:** 1 Department of Medical Biophysics, University of Toronto, Toronto, Ontario, Canada; 2 Physical Sciences Platform and Schulich Heart Research Program, Sunnybrook Research Institute, Toronto, Ontario, Canada; University of Illinois at Urbana-Champaign, UNITED STATES

## Abstract

To characterize and interpret arterial spin labeling (ASL) reactive hyperemia of calf muscles for a better understanding of the microcirculation in peripheral arterial disease (PAD), we present a physiological model incorporating oxygen transport, tissue metabolism, and vascular regulation mechanisms. The model demonstrated distinct effects between arterial stenoses and microvascular dysfunction on reactive hyperemia, and indicated a higher sensitivity of 2-minute thigh cuffing to microvascular dysfunction than 5-minute cuffing. The recorded perfusion responses in PAD patients (n = 9) were better differentiated from the normal subjects (n = 7) using the model-based analysis rather than characterization using the apparent peak and time-to-peak of the responses. The analysis results suggested different amounts of microvascular disease within the patient group. Overall, this work demonstrates a novel analysis method and facilitates understanding of the physiology involved in ASL reactive hyperemia. ASL reactive hyperemia with model-based analysis may be used as a noninvasive microvascular assessment in the presence of arterial stenoses, allowing us to look beyond the macrovascular disease in PAD. A subgroup who will have a poor prognosis after revascularization in the patients with critical limb ischemia may be associated with more severe microvascular diseases, which may potentially be identified using ASL reactive hyperemia.

## Introduction

**Fig 1:**
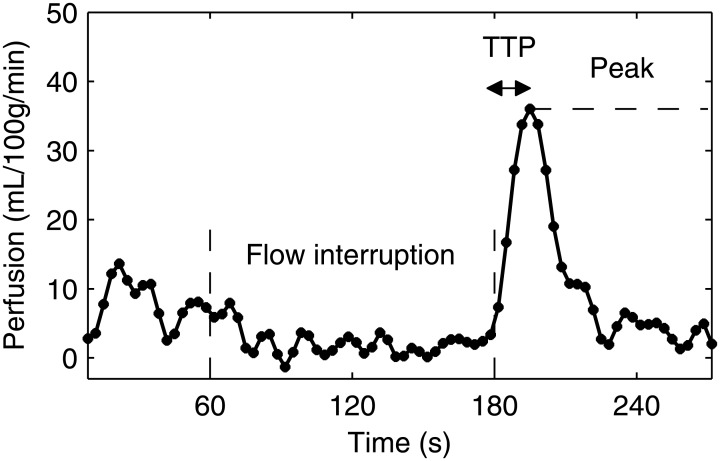
ASL reactive hyperemia induced by 2 minutes of flow interruption. TTP = time-to-peak.

Peripheral arterial disease (PAD) refers to the circulatory disorders in the leg as a consequence of peripheral atherosclerosis. Standard diagnostic techniques, such as the ankle-brachial index (ABI) and duplex ultrasound, have performed quite well for the detection of flow-limiting arterial stenoses. However, the influence of PAD is not confined to the conduit arteries but also affects the microvascular function of flow regulation and perfusion reserve [1]. Local perfusion in the tissue bed is dynamically controlled by the vascular smooth muscles of the arterioles, which adjust the wall tension in response to local vasoactive substances. While the standard techniques are poorly suited to assessing the microvascular function, a post-occlusive reactive hyperemia test, as illustrated in Fig 1, has been proposed. In this ischemia-reperfusion paradigm, the blood flow stopped by an inflated cuff would cause hypoxia and accumulation of metabolites in the tissue, leading to functional vasodilation and reduced flow resistance, such that the perfusion after the cuff is deflated can rapidly reach levels several folds higher than the resting condition. Arterial spin labeling (ASL) is a magnetic resonance imaging (MRI) technique that quantifies tissue perfusion in a spatially and temporally resolved fashion. With ASL, patients with PAD commonly demonstrate reduced and delayed reactive hyperemia in their calf muscles following a 5-minute cuffing [2–4].

However, using ASL reactive hyperemia to detect microvascular disease of PAD remains challenging. First, ASL reactive hyperemia is usually characterized by the peak and time-to-peak (TTP), which are determined based on a single data point of the perfusion response. Because the ASL response has low signal by nature, the noise has great influence on the variability of the two measured indices. Second, the exact vasodilatory mechanisms responsible for leg reactive hyperemia have not been fully understood, which limits the interpretation of abnormal responses observed in PAD. It is not clear if a blunted response implies arterial stenoses, microvascular dysfunction, or a combination of these effects. Lastly, the common 5-minute cuffing protocol was chosen in the past to induce large and prolonged reactive hyperemic arterial flow, for which the presence of flow-limiting stenoses would be highlighted. Given that the microvascular relaxation usually occurs tens of seconds after cuffing, increasing the sensitivity to microvascular dysfunction may require a different protocol.

The goal of this work is to provide the physiological basis of calf reactive hyperemia for data analysis and protocol establishment. We develop a subject-specific model to extract physiologically relevant features in individual responses. Our hypothesis is that a model-based analysis may help explain how the arterial stenoses and microvascular dysfunction affect the overall shape of the ASL reactive hyperemia response in the calf. The specific aims are: 1) to describe the tissue metabolic response during different cuffing durations up to 5 min; 2) to simulate reactive hyperemia under normal conditions and conditions of arterial stenoses and microvascular dysfunction; 3) to fit our acquired reactive hyperemia of cuffing duration-varying experiments in healthy subjects; and 4) to demonstrate the model’s potential of physiological interpretation in preliminary patient studies. If the aims are achieved, this study may lead to a more comprehensive assessment of limb ischemia and better classification of patient groups based on more than the macroscopic lesions in their arteries.

## The model

To deal with the challenges in characterization and interpretation of ASL data and standardization of the cuffing protocol, it is desirable to establish a model describing the essential physiological factors involved in reactive hyperemia. Flow models essentially describe the integrated effects of vascular segments connected in series, representing the arteries, microvasculature, and veins. Each vascular segment can be described with a combination of flow resistances (*R*) and vessel compliance (*C*). For dynamically regulated flow, the elements of resistance and compliance are made controllable to represent the microvascular responses to factors such as luminal pressure, shear stress, and vasoactive substances. Ursino et al. [5, 6] laid out a theoretical framework to link the physiological factors, including oxygen and metabolites, to the lumped circuit elements for cerebral blood flow regulation in various experimental conditions. However, the modeled reactive hyperemia was far off from experimental reality [7], mainly due to the lack of accurate characterization of the rapid vasodilatory response mechanisms. Adapting this framework, Spronck et al. [8] recently proposed a model suitable for fitting individual cerebral flow responses to experimental maneuvers. Meanwhile, Secomb’s group modeled the range of steady-state skeletal muscle perfusion with extensive study of metabolic flow regulatory mechanisms [9]. In addition, de Mul et al. [10] published a simple model which characterizes PAD patients’ laser Doppler reactive hyperemia with just two time constants, for the rise and decay of reactive hyperemia. Although the model did not take microvascular physiology into account, it demonstrated that a simple RC block is sufficient to characterize the effect of arterial stenoses on perfusion. Therefore, although a physiological model of skeletal muscle reactive hyperemia had not been established, there are plenty of reference materials for use as a starting point. We created the skeletal muscle perfusion model by adapting Spronck’s model and replacing the description of cerebral flow control with ischemia-induced vascular relaxation.

**Fig 2:**
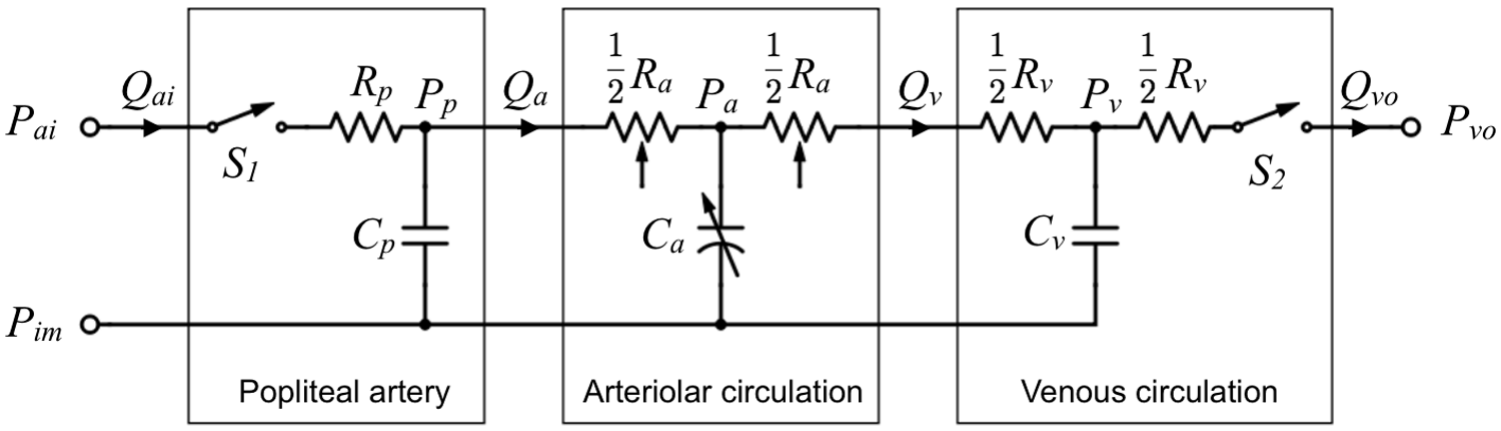
The flow circuit of calf circulation. *P*_*ai*_, *P*_*vo*_ and *P*_*im*_, denote arterial input, venous output, and intramuscular pressure, respectively. *Q*_*ai*_ and *Q*_*vo*_ denote popliteal arterial inflow and venous output flow, respectively. *Q*_*a*_ and *Q*_*v*_ are the flow to the arteriolar and venous circulation, respectively. The switches *S*_1_ and *S*_2_ represent the flow blocking effect of cuffing. *R*_*p*_: popliteal resistance, *C*_*p*_: popliteal compliance, *P*_*p*_: end arterial pressure for perfusion input to the arterioles; *R*_*a*_: arteriolar resistance, *C*_*a*_: arteriolar compliance, *P*_*a*_ mid-arteriole pressure; *R*_*v*_: venous resistance, *C*_*v*_: venous compliance, *P*_*v*_ mid-vein pressure.

A lumped parameter model of three vascular segments, shown in Fig 2, was used to describe the tibial circulation. Only the arteriolar segment contains adjustable elements, which regulate the flow through the entire modeled system. We solve the lumped parameters of the system as functions of time in order to simulate perfusion-time waveforms. The flow rate in each segment can be derived from Ohm’s law. Specifically, ASL perfusion *f*_*ASL*_ measured at the tissue level should correspond to *Q*_*v*_ in the model:

$$\begin{array}{c} f_{ASL}=Q_{v}/V_{m}=\frac{P_{a}-P_{v}}{(R_{a}+R_{v})/2} \end{array}$$ (1)

where the parameters are defined in Fig 2 caption. The steady-state baseline flow *Q*_*r*_ of the system is set to be the group average of our healthy subjects. The segmental pressure distribution of the system at *Q*_*r*_ is chosen to match the values in the references (see parameters in Table 1 where *P*_*im*_ was extracted from [11], *V*_*m*_ from [12], and the rest parameter values from [8]). The governing differential equations to describe the system’s response to flow interruption with time-varying lumped parameters will be derived.

**Table 1:** Constant parameters of the model.

Symbol	Value	Unit	Description
*P*_*vo*_	14	mmHg	Venous output pressure
*P*_*im*_	10	mmHg	Intramuscular fluid pressure
*Q*_*r*_	144	mL/min	Resting flow rate to the lower leg
*V*_*m*_	3	L	Estimated volume of one lower leg
*η*_*b*_	3×10^−5^	mmHg⋅s	Blood viscosity
*r*_*a*,0_	75	um	Inner radius of unstressed arteriolar wall
*h*_*a*,0_	25	um	Wall thickness of unstressed arteriole
*σ*_1_	11.19	mmHg	Constant for elastic tension model
*σ*_2_	52.51	mmHg	Constant for elastic tension model
*k*_*e*_	4.5	–	Constant for elastic tension
*T*_*m*,0_	3	mmHg⋅s	Maximal muscular tension of arterioles
*r*_*a*,*m*_	128	*μ*m	Radius at maximal muscular tension
*r*_*a*,*t*_	174	*μ*m	Constant for muscular tension model
*n*_*m*_	1.75	–	Constant for muscular tension model

### The lumped parameters

The popliteal artery is modeled as a simple resistor-capacitor unit. The normal arterial resistance is calculated assuming Poiseuille flow:

$$\begin{array}{c} R_{p}=\frac{8\eta_{b}}{\pi {r_{p}}^{4}}l_{p} \end{array}$$ (2)

where the arterial radius *r*_*p*_ = 0.25 cm and length *l*_*p*_ = 40 cm are estimated from population data, resulting in a resistance of 0.013 mmHg⋅min/mL and an arterial pressure drop *P*_*ai*_ − *P*_*p*_ = 1.8775 mmHg at *Q*_*r*_. The arterial capacitance *C*_*p*_ is chosen such that the arterial time constant matches the reported experimental values *τ*_*p*_ = *R*_*p*_*C*_*p*_ ≈ 5 s [10].

Venous resistance is chosen such that a venous pressure drop is *P*_*v*_ − *P*_*vo*_ = 1 mmHg at *Q*_*r*_. With an empirical choice of the venous time constant *R*_*v*_*C*_*v*_ = 10 s, *C*_*v*_ can be calculated. *R*_*v*_ and *C*_*v*_ are taken to be constant [8].

Because the arteriolar circulation is controlled by complex microvascular regulatory mechanisms rather than apparent lumped parameters with fixed values, it is necessary to express *R*_*a*_ and *C*_*a*_ with time-varying radius *r*_*a*_ and consider the relationship between the pressure and radius based on the mechanical properties of the arterioles [6, 8, 13]. In this regard, the mid arteriolar pressure *P*_*a*_ is related to the wall tension *T* and radius *r*_*a*_ by Laplace’s law. The wall tension consists of the passive elastic component *T*_*e*_ and active muscular component *T*_*m*_ scaled by the level of activation *A*. Together these give:

$$\begin{array}{c} T=P_{a}r_{a}-P_{im}\left(r_{a}+h_{a}\right)=T_{e}+AT_{m} \end{array}$$ (3)

where the arteriolar wall thickness *h*_*a*_ is formulated by:

$$\begin{array}{c} h_{a}=\sqrt{{r_{a}}^{2}+2r_{a,0}h_{a,0}+{h_{a,0}}^{2}}-r_{a}. \end{array}$$ (4)

The elastic tension is defined as:

$$\begin{array}{c} T_{e}=\left[\sigma_{1}e^{k_{e}\left(r_{a}-r_{a,0}\right)/r_{a,0}}-\sigma_{2}\right]\cdot h_{a} \end{array}$$ (5)

where constants are given in Table 1. The muscular tension generated by the fully activated arteriolar smooth muscle at a given radius is defined as:

$$\begin{array}{c} T_{m}=T_{m,0}e^{-|\frac{r_{a}-r_{a,m}}{r_{a,t}-r_{a,m}}{|}^{n_{m}}}. \end{array}$$ (6)

An arteriolar pressure drop Δ*P*_*a*_ = *P*_*p*_ − *P*_*v*_ = 70 mmHg and mid-arteriole pressure *P*_*a*_ = 50 mmHg are assumed for *Q*_*r*_ [14]. The resistance *R*_*a*_ and volume *V*_*a*_ (related to *C*_*a*_) expressed as a function of *r*_*a*_ is shown in Appendix A. The flow equations of the system.

With the lumped parameters defined, the governing equations of the flow system are expressed as:

$$\begin{array}{ccc} \frac{dP_{p}}{dt} & = & \frac{Q_{ai}-Q_{a}}{C_{p}}, \end{array}$$ (7)

$$\begin{array}{ccc} \frac{dr_{a}}{dt} & = & \frac{P_{a}\left(R_{v}-2R_{a}\right)+P_{p}\left(R_{a}-R_{v}\right)+P_{v}R_{a})}{r_{a}k_{V}R_{a}\left(R_{v}+Ra\right)},\;\text{and} \end{array}$$ (8)

$$\begin{array}{ccc} \frac{dP_{v}}{dt} & = & \frac{Q_{v}-Q_{vo}}{C_{v}}. \end{array}$$ (9)

### Microvascular regulation

The contribution of muscular tension to the total tension is adjusted by its’ activation level *A*, which is in the range [0, 1] and is defined as a sigmoidal function of the total regulatory influence *z*:

$$\begin{array}{c} A=\frac{1}{1+e^{-2z}}. \end{array}$$ (10)

The regulatory influences are explained in the following. At first, the myogenic and shear stress-based influences were considered because the blood pressure and flow are directly perturbed by thigh cuffing. Myogenic response refers to the reaction of vascular smooth muscle when the vessel is expanded by an increase in blood pressure. Shear stress is the drag force of viscous blood to the vessel wall. However, these two wall-derived mechanisms do not involve vasoactive substances. To date, the mechanisms specific for ischemia-induced vasodilation have not been clearly explained. Fortunately, evidence in the literature reveals that two microvascular regulatory mechanisms are very likely to be tied to ischemia-induced vasodilation. First, it is found that ATP released by red blood cells (RBCs) during hypoxia can activate the purinergic receptors on the endothelial cells and triggers endothelium-dependent vasorelaxation [15]. Second, ischemia-induced imbalance of energetic supply and demand would lead to an accumulation of interstitial adenosine, which can activate the receptors on vascular smooth muscles and induces endothelium-independent vasorelaxation [16]. Therefore, we defined the total regulatory influence as:

$$\begin{array}{c} z=\sum_{i}g_{i}x_{i}+x_{init} \end{array}$$ (11)

where i = [myo, sh, ATP, ado] stands for the myogenic, shear stress-based, intravascular ATP-mediated, and interstitial adenosine-mediated regulation, respectively. For each regulatory mechanism, the influence is the regulatory state *x* multiplied by the respective gain *g*. The parameter *x*_*init*_ sets the baseline arteriolar tension, radius, and resistance, which essentially determines the dynamic range for flow increases in the system.

The regulatory state is perturbed by the associated stimulus and varies based on a certain time constant (via a first-order approach):

$$\begin{array}{c} \frac{dx_{i}}{dt}=\frac{y_{i}-x_{i}}{\tau_{i}} \end{array}$$ (12)

where *τ*_*i*_ stands for the time constant of each regulatory mechanism. The definition of the stimulus function *y*_*i*_ is explained in Appendix B. Modeling the regulatory influences. In short, *y*_*myo*_ is the deviation of current arteriolar wall tension from the baseline tension, *y*_*sh*_ the deviation of current shear stress from the baseline shear stress, and so on. Since the wall-derived regulatory mechanisms have been well characterized and are known to be relatively weak when compared with metabolic flow regulation [9], we chose to use fixed values similar to a previous study [8]: *g*_*myo*_ = 1, *g*_*sh*_ = 1, *τ*_*myo*_ = 6 s, and *τ*_*sh*_ = 60 s.

We assumed that intravascular ATP and interstitial adenosine are the main vasoactive substances for metabolic regulations because their production depends on the level of oxygenation in the capillary and tissue, respectively. Therefore, a two-compartment model for the transport of oxygen is required. We adapted Lai’s model and used a constant permeability surface area-product (*D*) for diffusion of oxygen between the capillary and tissue compartment [17]. The differential equations of compartmental oxygen pressure are:

$$\begin{array}{ccc} \frac{dPO_{2c}}{dt} & = & \frac{f\left(O_{2art}-O_{2c}\right)-\alpha D\left(PO_{2c}-PO_{2t}\right)}{v_{c}\gamma_{c}} \end{array}$$ (13)

$$\begin{array}{ccc} \frac{dPO_{2t}}{dt} & = & \frac{\alpha D\left(PO_{2c}-PO_{2t}\right)-VO_{2}}{v_{t}\gamma_{t}} \end{array}$$ (14)

where *PO*_2*c*_ and *PO*_2*t*_ are the oxygen partial pressure of the capillary and tissue compartment, respectively. *O*_2*art*_ and *O*_2*c*_ are the oxygen concentration of the inflow arterial and capillary blood, respectively. The solubility of oxygen in blood *α* is 1.46 *μ*M/mmHg, and volume fraction of capillary *v*_*c*_ and tissue *v*_*t*_ are 3% and 97%, respectively. The metabolic function *VO*_2_ and the parameter *γ* = *dO*_2_/*dPO*_2_, varying with the level of oxygenation, are defined in Appendix B. Modeling the regulatory influences.

The concentration of intravascular ATP *C*_*ATP*_ can be calculated by considering the inflow arterial ATP concentration *C*_*ATP*,*in*_, oxygen saturation-dependent ATP release rate R, and degradation by the endothelial surface:

$$\begin{array}{c} \frac{dC_{ATP}}{dt}=\frac{f}{v_{c}}\frac{1-h_{d}}{1-h_{t}}\left(C_{ATP,in}-C_{ATP}\right)+\frac{h_{t}}{1-h_{t}}R-\frac{2k_{d}}{r_{c}\left(1-h_{t}\right)}C_{ATP} \end{array}$$ (15)

where *C*_*ATP*,*in*_ = 0.1 *μ*M, the capillary radius *r*_*c*_ = 3*μ*m, tube hematocrit *h*_*t*_ = 0.4, discharge hematocrit *h*_*d*_ = 0.3, and degradation constant *k*_*d*_ = 2 × 10^−4^ cm/s [9]. The ATP release function R is given by in Arciero’s [9]:

$$\begin{array}{c} R\left(PO_{2c}\right)=R_{0}\left(1-R_{1}\frac{{PO_{2c}}^{n}}{{PO_{2c}}^{n}+{P_{50,Hb}}^{n}}\right) \end{array}$$ (16)

where *R*_0_ = 84 *μ*M/min, *R*_1_ = 0.891, *P*_50,*Hb*_ = 26.8 mmHg, and *n* = 2.7.

As ischemia progresses in the tissue compartment during cuffing, the skeletal muscle switches from aerobic metabolism to anaerobic metabolism and the formation of interstitial adenosine *C*_*ado*_ is increased. On the other hand, interstitial adenosine is cleared slowly by cellular uptake back to the tissue with the rate described by Michaelis-Menten equation [18]. Therefore, the dynamic of *C*_*ado*_ is formulated as:

$$\begin{array}{c} \frac{dC_{ado}}{dt}=F_{ado}-V_{max,ado}\frac{C_{ado}}{C_{ado}+K_{m,ado}} \end{array}$$ (17)

where *V*_*max*,*ado*_ = 100 *μ*M/min and *K*_*m*,*ado*_ = 200 *μ*M are chosen for the adenosine clearance rate [19]. By referring to the concentration in various physiological conditions [20, 21], the adenosine formation rates *F*_*ado*_ in *μ*M/min are assumed here to be:

$$\begin{array}{ccc} F_{ado}\left(PO_{2t}\right) & = & 0.05\;PO_{2t}\geq PO_{2cr} \end{array}$$ (18)

$$\begin{array}{ccc} & = & 0.5\;\;\text{otherwise} \end{array}$$ (19)

where the critical oxygen pressure *PO*_2*cr*_ for anaerobic metabolism is assumed to 10 mmHg [22]. The baseline interstitial concentration of adenosine is 0.1 *μ*M.

### Model behavior

Simulation of ischemia and reperfusion under normal and diseased conditions is presented in this section to facilitate interpretation of the model behavior. This simulation was developed based on a set of standard parameters reflecting the mean response of the healthy group, as described later in the methods and results sections.

**Fig 3:**
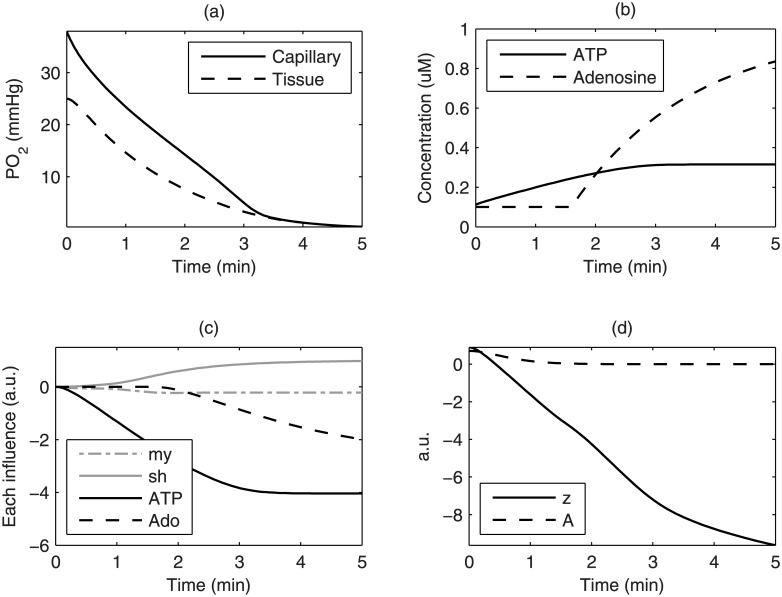
Ischemic responses of the tissue and arterioles. Modeling the responses during 5 minutes of ischemia in the muscle, including (a) the oxygen tension, (b) concentrations of the vasoactive substances, (c) the influences of vasoregulation mechanisms, and (d) the summed influence *z* and level of activation *A* of vascular smooth muscle.

During ischemia, the muscle tissue continues to consume oxygen at the baseline metabolic rate until the *PO*_2_ is relatively low, as simulated in Fig 3a. The concentration of intravascular ATP increases with deoxygenation in the capillary compartment, whereas the interstitial adenosine only starts to increase when *PO*_2*cr*_ is reached and anaerobic metabolism begins, as delineated in Fig 3b. The influences of each regulatory mechanism are drawn in Fig 3c. While the total regulatory influence *z* continues to be enhanced by the accumulation of metabolites, the vascular smooth muscle is almost de-activated after 2 minutes of ischemia, as shown by the level of activation *A* in Fig 3d.

**Fig 4:**
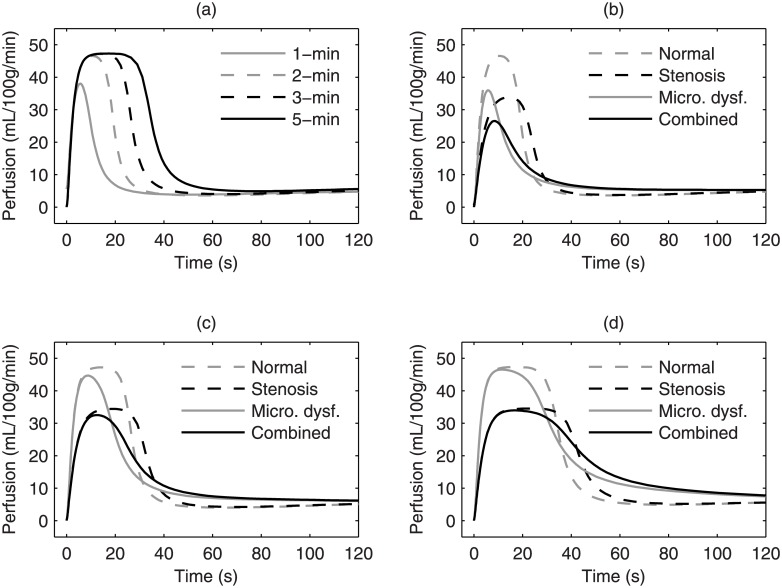
The effects of ischemic duration, arterial stenosis, and microvascular dysfunction on reactive hyperemia. (a) Normal responses to various ischemic durations and diseased responses to (b) 2-min, (c) 3-min, and (d) 5-min of ischemia. Arterial stenosis was simulated by doubling the popliteal resistance *R*_*p*_, and microvascular dysfunction was simulated by reducing *g*_*ATP*_ to 40%. “Combined” includes both effects at the same time.

The modeled reactive hyperemia is shown in Fig 4. Because of the rapid nature of intravascular ATP-mediated vasorelaxation, an evident response can be induced with just one minute of ischemia. As illustrated in Fig 4a, increasing ischemic duration would slightly enhance the magnitude of the modeled response, but ischemia longer than 2 minutes would mostly prolong the modeled reactive hyperemia. Arterial stenoses simulated by doubling *R*_*p*_ would reduce the response magnitude regardless of ischemic duration, as depicted in Fig 4b, 4c and 4d, because it reduces the input perfusion pressure to the microcirculation. Doubled *R*_*p*_ would also double the arterial time constant, resulting in longer rise time. Microvascular dysfunction does not delay the rise time, on the other hand. Depending on the ischemic duration used, microvascular dysfunction does not necessarily reduce the magnitude of the responses to a large extent. As simulated by reducing *g*_*ATP*_ in the model, microvascular dysfunction appears to cause earlier attenuation, suggesting that the dysfunctional arterioles tend to constrict. When comparing microvascular dysfunction alone with normal function, or combined micro- and macro-vascular disease with stenoses alone, the differences are more evident in the responses to 2-min ischemia than longer ischemia. This result of modeling may be explained by the fact that as the concentration of interstitial adenosine increases in longer ischemia the vascular smooth muscles eventually relax, regardless of the endothelium-dependent ATP signal. Therefore, the model suggests that a protocol using shorter ischemic duration may be more sensitive to endothelium-related microvascular dysfunction.

## Materials and methods

### Subjects and data acquisition

The perfusion data was acquired previously in a separate work of sequence optimization, and a portion of it was summarized in S1 Supplementary Material. In brief, the study included the mid-calf ASL reactive hyperemia of 7 healthy subjects (age <30 yrs) and 9 patients with PAD. All of the healthy subjects underwent 2, 3, and 5 minutes of arterial occlusion via cuff inflation (220–250 mmHg); five of them also participated in a 1-min arterial occlusion experiment. Each of the patients only underwent 2-min cuffing for the more affect leg, which was determined based on self-reported symptoms and physicians’ dictation. The median age of the patient group was 74.5 yrs (51 to 83). The ABI ranged from 0.3 to 0.87. Seven patients had claudication, and the remaining two had rest pain.

The ASL technique was flow-sensitive alternating inversion recovery (FAIR) with single-slice steady-state free precession for acquisition, performed at 3T (MR 750 by GE, Milwaukee, Wisconsin) with an 8-channel cardiac receive array placed in the calf region. Sequence parameters were: field of view = 16 cm × 16 cm, matrix size = 64×64, slice thickness = 1 cm, single-slice steady-state free precession (SSFP) acquisition, flip angle = 70°, post-labeling delay (PLD) = 1.4 s, bolus duration *TI*_1_ = 700 ms. Image acquisition was repeated every 3.35 s. The images were reconstructed and processed in MATLAB. Regions of interest were manually drawn to enclose the entire muscle area excluding the large vessels. The difference signal Δ*M*(*t*) was converted to perfusion *f*(*t*) as follows:

$$\begin{array}{c} f_{ASL}\left(t\right)=\frac{\lambda }{2TI_{1}}\frac{\Delta M(t)}{M_{0b}}e^{-PLD/T_{1b}} \end{array}$$ (20)

where *M*_0*b*_ is the signal of fully relaxed blood, *T*_1*b*_ the blood longitudinal relaxation time, and the blood volume partition coefficient λ = 0.9mL/g. Reactive hyperemia was characterized by the peak and TTP.

### Modeling and fitting

The flow regulation system described previously, including the lumped parameters and oxygen transport model, was implemented using MATLAB (see S1 Model File, the entire model called RHmodel). Modeled reactive hyperemia was generated by solving the governing differential eqs (7)–(9) and (12), using the built-in solver function *ode15s*. Subject-specific model parameters were identified as described in the next two paragraphs. The values of these parameters were estimated by searching for the minimum least-squares fit between the modeled and ASL-measured perfusion, using the built-in optimization function *lsqnonlin*. The quality of fit was assessed by calculating the reduced chi-square:

$$\begin{array}{c} \chi_{red}^{2}=\frac{1}{N-n-1}\sum_{i}^{N}\frac{{\left(f\left(i\right)-f_{ASL}\left(i\right)\right)}^{2}}{\sigma^{2}} \end{array}$$ (21)

where *σ*^2^ is the variance of baseline perfusion, N the data length, and n the number of free parameters.

For the healthy group, [*f*_*r*_, *g*_*ATP*_, *τ*_*ATP*_, *g*_*ado*_, *τ*_*ado*_, *τ*_*p*_] were chosen as the free parameters. The logic lies in the approach to address the individual differences in the resting perfusion *f*_*r*_ (= *Q*_*r*_/*V*_*m*_) and reactive hyperemia. First, we attributed the variation of resting perfusion in healthy individuals to the baseline arteriolar tone, reflecting the variation in capillary permeability and tissue metabolism, not the variation in resistances of the arteries or veins. Therefore, *f*_*r*_ was varied to determine *x*_*init*_ for the baseline arteriolar tone. The group average resting perfusion was aligned with a positive *x*_*init*_ = 0.45 chosen empirically. Next, we assumed that the differences in reactive hyperemia result from the variation in the sensitivities and time constants of ATP and adenosine in addition to the arterial time constant (reflecting the difference in arterial compliance if the resistance is fixed). Therefore, the steps of fitting the cuffing duration-varying reactive hyperemia are: 1) use [*f*_*r*_, *g*_*ado*_, *τ*_*ado*_, *τ*_*p*_] with fixed *g*_*ATP*_ and *τ*_*ATP*_ to fit the responses to 3- and 5-min cuffing; and 2) use [*f*_*r*_, *g*_*ATP*_, *τ*_*ATP*_] with the resulting *g*_*ado*_, *τ*_*ado*_, and *τ*_*p*_ from step 1 to fit the responses to 1- and 2-min cuffing. The parameters *g*_*ATP*_ and *τ*_*ATP*_ were not included as free parameters in step 1 because their influences on the response to long cuffing were extremely small and a wide range of their values can be used, making the resulting values unstable and not meaningful. Likewise, only the more sensitive parameters for short cuffing were included in step 2. The allowed range of free parameters in the fitting process are: 2 ≤ *f*_*r*_ ≤ 10 (mL/100g/min), 1 ≤ *g*_*ATP*_ ≤ 20, 1 ≤ *g*_*ado*_ ≤ 15, 6 ≤ *τ*_*ATP*_ ≤ 24 (s), 12 ≤ *τ*_*ado*_ ≤ 60 (s), 1.5 ≤ *τ*_*p*_ ≤ 30 (s).

For the patient group, [*f*_*r*_, *g*_*ATP*_, *τ*_*ATP*_, *R*_*p*_, *τ*_*p*_] were chosen as the free parameters. *R*_*p*_ was included as a free parameter because these PAD patients have various degrees of arterial stenoses affecting reactive hyperemia. Adenosine parameters were not included as free parameters because of their minimal influence on perfusion response to 2-min cuffing. Their values were fixed to the average values of the healthy group. The allowed range of free parameters in the fitting process are: 3 ≤ *f*_*r*_ ≤ 8 (mL/100g/min), 1 ≤ *g*_*ATP*_ ≤ 12, 12 ≤ *τ*_*ATP*_ ≤ 36 (s), 1.5 ≤ *τ*_*p*_ ≤ 25 (s), and 1 ≤ *R*_*p*_ ≤ 6 (starting from here *R*_*p*_ is expressed as the ratio to the calculated standard value from Eq (2)). The ranges of these parameters were narrower than those used in the healthy group because the physiological ranges had been known after fitting the healthy responses.

### Statistical analysis

The peak, TTP, and model parameters that resulted in the best fit to the responses were compared between the healthy subjects and patients using an unpaired *t*-test. A *p*-value of 0.05 was assumed to indicate statistical significance.

## Results

**Fig 5:**
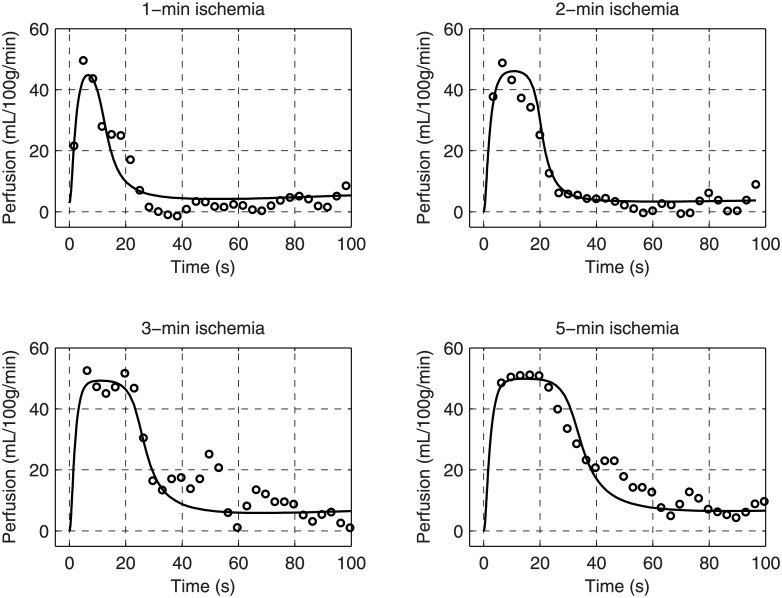
Fitting the responses of a healthy subject with the model. The resulting parameters are: [*f*_*r*_, *g*_*ATP*_, *g*_*ado*_, *τ*_*ATP*_, *τ*_*ado*_, *τ*_*p*_] = [5.59, 14.67, 3.49, 18.0, 44.5, 3.05].

The model could generate ischemic-duration-dependent responses very similar to the measured reactive hyperemia in healthy subjects. The model achieved reasonable fits with one set of parameters for all responses to various durations of cuffing in each subject, as shown in Fig 5. The quality of fit, measured by $\chi_{red}^{2}$, was affected by baseline drift, sudden jumps, and small oscillations of the ASL signal in some cases. Noise level varied between recordings. Looking through the healthy group, the model did not necessarily fit better to the large and long responses (5-min cuffing) than the transient responses.

The parameter values of the healthy group were summarized as means ± standard deviations: *f*_*r*_ = 4.95 ± 0.92 mL/100g/min, *g*_*ATP*_ = 15.66 ± 2.05, *g*_*ado*_ = 4.18 ± 2.41, *τ*_*ATP*_ = 15.86 ± 2.66 s, *τ*_*ado*_ = 43.20 ± 21.11 s, and *τ*_*p*_ = 4.25 ± 1.16 s. The parameter values and $\chi_{red}^{2}$ of each fit were reported in S1 Dataset. The parameters related to adenosine had a broader range than other parameters, with coefficients of variation (CV) 27.4% and 49.6% for *g*_*ado*_ and *τ*_*ado*_, respectively. The rest of parameters were relatively consistent within the group (CV<20%).

**Fig 6:**
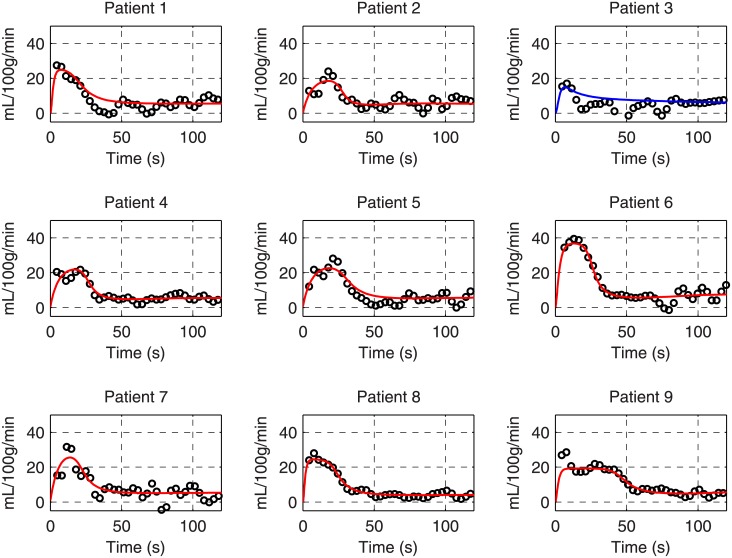
Fitting of patients’ responses to 2-min cuffing with the model-generated response. The fitting with exceptionally large error is plotted as the blue line.

The model fitting of diseased responses to 2-min cuffing was shown in Fig 6. Eight out of the 9 responses had $\chi_{red}^{2}$ ranged between 0.59 to 1.39, indicating reasonable fits; response 3 had a $\chi_{red}^{2}$ of 4.17, indicating a poor fit (see S1 Model File for each fitting). Each recording had a different level of noise. Response 3 was very small and only three data points were two standard deviations above the baseline perfusion, which was not sufficient to determine the five free model parameters.

**Fig 7:**
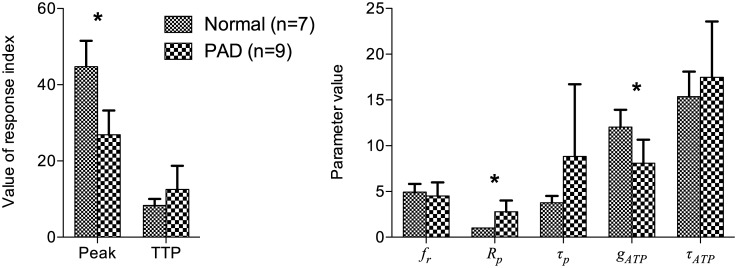
Characteristics of reactive hyperemia induced by 2-min cuffing. *Left*: characterization using apparent indices. *Right*: characterization using model-derived parameters. * denotes statistical significance (P < 0.05).

The model parameters were more variable within the patient group than the healthy group. As compared in Fig 7, responses in the patient group were significantly lower in peak perfusion, but not in TTP (*p* = 0.102), than those in the healthy group. With the model-based analysis through fitting, the patient group showed higher *R*_*p*_ and lower *g*_*ATP*_ than the healthy group. The *p*-values were 0.285, 0.098, and 0.461 for comparison of *f*_*r*_, *τ*_*p*_, and *τ*_*ATP*_.

## Discussion

### Establishment of the model

We sought to provide an explanation to the dependence of reactive hyperemic characteristics on ischemic duration using the model. Although reactive hyperemia is often related to endothelial function, which is commonly assessed by flow-mediated dilatation via the effect of shear stress, it is unlikely that shear stress plays a significant role in ischemia-induced vasodilation. The first reason is that flow interruption should lead to shear-dependent vasoconstriction, not vasodilation. Second, the change in shear stress induced by cuffing is independent of the ischemic duration and hence should not enhance the perfusion response if ischemic duration increases. The same arguments can be applied to the pressure-related myogenic regulatory mechanism. Indeed, it has been suggested that these wall-derived mechanisms should be relatively weak and work against the metabolic flow regulation [9]. To identify the mechanisms more relevant to ischemia, we searched for mechanisms closely tied to the dynamics of the deoxygenation process during ischemia. Studies of magnetic resonance spectroscopy have shown that myoglobin desaturation begins at 100–120 s and plateaus at 300–420 s in the course of acute ischemia [23, 24]. The initial delay of myoglobin desaturation in the tissue pool is due to the contribution of oxygen from the blood pool. At the later stage of ischemia, high level of myoglobin desaturation corresponded to the onset of anaerobic metabolism indicated by the change of phosphocreatine in the tissue. These two-compartment deoxygenation dynamics were incorporated in our model (Fig 3a) to control the metabolite-derived regulatory mechanisms.

In the capillary compartment, we linked vascular tension to the oxygen saturation-dependent release of ATP by RBCs. The theory of RBCs being the oxygen sensor for local flow regulation has long been established [15]. Our simulated concentration of ATP was in the submicromolar range, consistent with intravascular measurements in human hypoxia experiments [25]. The estimated time constant of ATP-mediated regulation *τ*_*ATP*_ was 15.4 ± 2.7 s, which is very close to the reported values (8–16 s) from direct observation of exposed arterioles in an animal study [26]. The function of the ATP receptor on the endothelium was found to be attenuated in type 2 diabetes [27]. This finding corresponded to a reduction in *g*_*ATP*_ in our model and was simulated as microvascular dysfunction. Consistent with the simulation, reduced *g*_*ATP*_ was found in the patient group.

In the tissue compartment, the concentration of interstitial adenosine was modeled. Increased formation of adenosine during hypoxia results from the elevated activity of ecto 5’-nucleotidase converting muscle-released AMP into adenosine [28]. However, it is difficult to quantify the exact reaction rate and concentrations of AMP and adenosine because a wide range of values was reported, potentially because real-time interstitial measurements *in vivo* remain challenging [29]. Hence, we only roughly estimated the adenosine formation rate and described it as a simple 2-mode function in Eq (18). The high variability of *g*_*ado*_ and *τ*_*ado*_ in the fitting of normal responses (Fig 6) may result from such simplification of adenosine formation. Furthermore, other metabolites, such as lactic acid and hydrogen ions, may also be involved in the tissue compartment for extended ischemia. Therefore, the current model may be less accurate in explaining/simulating the responses to longer ischemia.

With intravascular ATP and interstitial adenosine incorporated, the model clearly demonstrated ischemic duration-dependent responses. The rapid magnitude increase for responses to short ischemia is very likely to be contributed by the ATP released by deoxygenated RBCs. On the other hand, longer ischemia appeared to prolong the responses by accumulating adenosine in the tissue. The relationship of the peak and TTP on ischemic duration in the modeled reactive hyperemia agreed with the previously measured characteristics in the healthy group. From a statistical perspective, however, some of the acquired responses did not entirely agree with the model. ASL data may contain not just metabolic perfusion response but also imaging noise, motion artifacts introduced by cuff inflation and deflation, and higher order vessel properties causing flow oscillation during recovery. These details were not considered in the model, which may be the reason for poor fitting in some cases. Previously, Spronck’s model was used to fit individual cerebral flow response averaged by repeated recordings, which presumably decreased the experimental variability associated with these influences. Therefore, our model may fit better to averaged individual responses, which should be more representative of metabolic flow responses. The fitting may be very likely to fail when the diseased response is very small. However, cases with such small responses may essentially indicate severe microvascular disease and model analysis is not required.

### Major findings

An important contribution of this work is that the model delineates the different effects of arterial stenoses versus microvascular dysfunction on reactive hyperemia. Arterial stenoses can limit response peak to a great extent. However, there are already many tools to assess arterial lesions. The use of ASL reactive hyperemia was motivated by the hope to look at microvascular function. According to our simulation, a shorter ischemic duration can better detect microvascular dysfunction. This finding reflects the often overlooked fact that the microvasculature typically reacts quickly to ischemia and a short ischemic protocol can be used to measure the quickness of reactivity. To date, 5-min cuffing is recommended for reactive hyperemia measurement due to its lower variability in the peak and TTP when compared with shorter cuffing. From a signal perspective, our simulation agrees with this viewpoint that the 2-min cuffing response may be too short to provide enough data points to measure the exact peak difference caused by arterial lesions. However, our model simulation suggested that the peak and TTP are more useful for detecting the influence of macroscopic lesions rather than quantifying microvascular dysfunction. The model clearly indicated that the unique potential of ASL reactive hyperemia for microvascular assessment is highlighted when using 2-min cuffing. Therefore, we proposed to use a 2-min ischemic protocol for more sensitive microvascular assessment.

With 2-min cuffing, the PAD group demonstrated lower peaks and slightly longer TTP than the healthy group (Fig 7), which is similar to the previous findings using 5-min cuffing [4]. A significant difference in TTP may be found if subject size increases. Another strategy to raise significance is to reduce variability by taking model fitted peak and TTP, since the raw peak and TTP is based on a single data point so is highly susceptible to noise. Fitting the responses to a known mathematical function, such as a gamma-variate function, has been demonstrated for reactive hyperemic blood oxygen-level dependent imaging [30]. While the function may also fit ASL reactive hyperemia of the healthy subjects, the diseased responses may not resemble a gamma-variate function, as exemplified by response 9 in Fig 6. More importantly, an empirical function does not contain physiological meaning, let alone explaining the dependence of reactive hyperemia on cuffing duration. With our model-derived analysis, we found that some of the model parameters not only clearly differentiate the responses of the two groups but may also suggest direct pathophysiological implications. First, the increased *R*_*p*_ may represent arterial stenoses. Second, the arterial time constant *τ*_*p*_ is a function of both resistance and compliance. Since the two factors are not necessarily tightly correlated and have the opposite effect on *τ*_*p*_ as they progress with PAD, a wide range of values in patients is not surprising. Lastly, the *g*_*ATP*_, representing the microvascular endothelial sensitivity to ATP in our model, was significantly lower overall in patients but also had greater variation within the patient group. This result suggests that different levels of microvascular disease exist within the patient group and may be assessed by ASL reactive hyperemia with our model-based analysis. The variation in the microvascular parameter may have critical clinical implications.

It is known that a large portion of the patients with critical limb ischemia does not achieve limb salvage 1 year after revascularization, which could be caused by more advanced microvascular diseases. This work strongly demonstrates the potential of using ASL reactive hyperemia for microvascular assessment in such a patient population, which may be useful to identify the subgroup who requires more effective treatment for their microvascular disease.

The scope of this work was determined to focus on the mathematics and physiological concepts about reactive hyperemia. As a first step, we only explored the perfusion characteristics in healthy subjects and patients with known disease. Testing the clinical relevance of the derived perfusion indices will be the next focus. The perfusion measure for the arterial resistance can be tested by a comparison between the more and less affected leg of the same patient. A bilateral comparison can exclude the variability caused by the differences in subjects’ cardiac output, blood pressure, blood viscosity, and other systematic factors, and the results may be more meaningful than a comparison between patients and age-matched non-PAD subjects. On the other hand, a standard microvascular assessment that can be used to validate the perfusion measures of microvascular function does not yet exist. However, the relevance of the microvascular measures to the outcome can be tested by a post-revascularization prognostic study.

So far, the model only explained the data from small subject groups. With more data accumulated, the patients can be stratified based on their clinical indications, and the perfusion indices between subgroups can be compared. Also, the dependence among model-derived parameters and the correlation between the parameters and clinical indices may be further examined. The model description of reactive hyperemia should not be generalized for exercise-induced active hyperemia, as capillary recruitment in exercise can greatly change the permeability surface area-product. Since the heterogeneity between calf muscle groups was not the focus here, perfusion of the whole mid-calf cross section was used for the establishment of the model. Using perfusion data from smaller ROIs with higher noise in ASL does not help extract consistent physiology for a general description of the response to ischemia. Although the model fitted reasonably well to our data, the quality of ASL data remains critical. Large SNR and minimum motion artifact can reduce bias in the fitting. The experiments of 2-min thigh cuffing were well tolerated by patients, but repeated recording could be affected by previous ischemia, especially in patients with reduced washout rate of metabolites.

## Conclusion

We established a detailed physiological model to provide a description of the flow regulatory mechanisms involved in ASL reactive hyperemia of calf skeletal muscles. The model demonstrated that combining the dynamics of intravascular ATP released by RBCs and interstitial adenosine can explain the evolution of response characteristics with increasing durations of cuffing in healthy subjects. Model simulation suggested influences of microvascular dysfunction distinct from those of arterial stenoses on reactive hyperemia. Microvascular dysfunction may be detected more easily in a 2-min cuffing protocol rather than longer cuffing, whereas the effect of arterial stenoses may be more consistently observed using 5-min cuffing. Model-based analysis of the patient data showed that *R*_*p*_ and *g*_*ATP*_ were significantly different between the patient and healthy group. While increased *R*_*p*_ in patients is normally expected, the variation of *g*_*ATP*_ within the patient group may suggest that different amounts of microvascular disease existed. Therefore, we propose next to test the utility of ASL reactive hyperemia and our model-based analysis in the patients with critical limb ischemia who require assessment of their microcirculation in addition to their arterial lesions.

### Selected symbols

*f*_*r*_: Resting perfusion; *g*_*ATP*_: Gain of intravascular ATP-mediated regulation; *g*_*ado*_: Gain of interstitial adenosine-mediated regulation; *R*_*p*_: Popliteal artery resistance; *τ*_*ado*_: Response time to adenosine; *τ*_*ATP*_: Response time to ATP; *τ*_*p*_: Popliteal artery inflow time constant; $\chi_{red}^{2}$: Reduced chi-square.

## Appendix

### A. The flow equations of the system

The arteriolar resistance *R*_*a*_ and volume *V*_*a*_ are defined as:

$$\begin{array}{ccc} R_{a} & = & K_{R}/{r_{a}}^{4} \end{array}$$ (22)

$$\begin{array}{ccc} V_{a} & = & K_{V}{r_{a}}^{2} \end{array}$$ (23)

where the constants *K*_*R*_ and *K*_*V*_ are chosen such that at *r*_*a*_ = *r*_*a*,0_ (baseline), *R*_*a*_ = Δ*P*_*a*_/*Q*_*r*_, *R*_*a*_*C*_*a*_ = 1 s, and *V*_*a*_ = *C*_*a*_Δ*P*_*a*_.

The arterial flow eq (7) is derived from the definition of compliance being a change of volume due to pressure change:

$$\begin{array}{ccc} C_{p} & = & \frac{dV_{p}}{dP_{p}}=\frac{dV_{p}}{dt}\frac{dt}{dP_{p}} \end{array}$$ (24)

$$\begin{array}{ccc} \frac{dV_{p}}{dt} & = & Q_{ai}-Q_{a} \end{array}$$ (25)

where *V*_*p*_ is the volume of the popliteal artery. The venous flow eq (9) is derived likewise.

The arteriolar flow eq (8) is derived by taking the time derivative of Eq (23) and expressing the volume change as the difference between the input *Q*_*a*_ and output flow *Q*_*v*_:

$$\begin{array}{ccc} \frac{dV_{a}}{dt} & = & K_{V}r_{a}\frac{dr_{a}}{dt}=Q_{a}-Q_{v} \end{array}$$ (26)

$$\begin{array}{ccc} Q_{a} & = & \frac{P_{p}-P_{a}}{2R_{a}} \end{array}$$ (27)

$$\begin{array}{ccc} Q_{v} & = & \frac{P_{a}-P_{v}}{2(R_{a}+R_{v})} \end{array}$$ (28)

### B. Modeling the regulatory influences

The stimulus functions of wall-derived regulatory mechanisms are taken from Spronck’s model [8]. The myogenic stimulus is calculated based on the deviation of arteriolar wall tension *T* from the baseline *T*_0_, multiplied by a scaling factors *s*_*my*_:

$$\begin{array}{c} y_{my}=s_{my}\left(T-T_{0}\right) \end{array}$$ (29)

where *s*_*my*_ = 3.33 (mmHg⋅cm)^−1^.

The shear stress is proportional to *Q*/*r*^3^. Therefore, the shear stress stimulus is defined as:

$$\begin{array}{c} y_{sh}=s_{sh}\frac{Q_{v}}{{r_{a}}^{3}}-1 \end{array}$$ (30)

where *s*_*sh*_ is chosen such that at rest the baseline flow and arteriolar radius will result in zero stimulus.

Likewise, the stimulus functions of metabolite-derived regulatory mechanisms, including the intravascular ATP and extravascular adenosine, are defined as the deviation from the baseline concentrations *C*_*ATP*,0_ and *C*_*ado*,0_ multiplied by scaling factors:

$$\begin{array}{ccc} y_{ATP} & = & s_{ATP}\left(C_{ATP}-C_{ATP,0}\right) \end{array}$$ (31)

$$\begin{array}{ccc} y_{ado} & = & s_{ado}\left(C_{ado}-C_{ado,0}\right) \end{array}$$ (32)

where *s*_*ATP*_ and *s*_*ado*_ are empirically chosen to be 2.5 and 0.61 *μ*M^−1^, respectively.

The concentrations of oxygen in the capillary *O*_2*c*_ and tissue compartment *O*_2*t*_ are the sums of freely dissolved oxygen and oxygen bound to hemoglobin and myoglobin, respectively:

$$\begin{array}{ccc} O_{2c}\left(PO_{2c}\right) & = & 4C_{Hb}\frac{{PO_{2c}}^{n}}{{PO_{2c}}^{n}+{P_{50,Hb}}^{n}}+w_{b}\alpha PO_{2c} \end{array}$$ (33)

$$\begin{array}{ccc} O_{2t}\left(PO_{2t}\right) & = & C_{Mb}\frac{PO_{2t}}{PO_{2t}+P_{50,Mb}}+w_{t}\alpha PO_{2t} \end{array}$$ (34)

where *C*_*Hb*_ = 2.33mM is the concentration of hemoglobin, *C*_*Mb*_ = 365*μ*M the concentration of myoglobin, *w*_*b*_ = 80.95% the fraction of water in blood, and *w*_*t*_ = 78% the fraction of water in the tissue. The parameters *γ* = *dO*_2_/*dPo*_2_ for Eqs (13) and (14) are:

$$\begin{array}{ccc} \gamma_{c} & = & 4C_{Hb}\frac{n{P_{50,Hb}}^{n}{PO_{2c}}^{n-1}}{{\left({PO_{2c}}^{n}+{P_{50,Hb}}^{n}\right)}^{2}}+w_{b}\alpha \end{array}$$ (35)

$$\begin{array}{ccc} \gamma_{t} & = & C_{Mb}\frac{P_{50,Mb}}{Po_{2t}+P_{50,Mb}}+w_{t}\alpha \end{array}$$ (36)

With *O*_2*t*_ defined, the skeletal muscle metabolic function is calculated:

$$\begin{array}{c} VO_{2}\left(O_{2t}\right)=V_{max,m}\frac{O_{2t}}{O_{2t}+K_{m,O_{2}}} \end{array}$$ (37)

where *K*_*m*,*O*_2__ = 0.7 *μ*M, and *V*_*max*,*m*_ is calculated from the individual baseline perfusion.

The change of intravascular ATP concentration Eq (15) is derived by considering the total amount of ATP in a single capillary with inflow, release and degradation:

$$\begin{array}{c} \pi {r_{c}}^{2}l_{c}\left(1-h_{t}\right)\frac{dC_{ATP}}{dt}=q\left(1-h_{d}\right)\left(C_{ATP,in}-C_{ATP}\right)+\pi {r_{c}}^{2}l_{c}h_{t}R-2\pi r_{c}l_{c}k_{d}C_{ATP} \end{array}$$ (38)

where the capillary length *l*_*c*_, flow rate of a single capillary *q*. Since capillary flow can be derived from perfusion and capillary volume fraction, this equation can be simplified into Eq (15).

## Supporting information

S1 Model File RHmodel and perfusion data to produce Figs 3, 4, 5 and 6. This zip-file contains functions and perfusion data in MATLAB’s format.
(ZIP)

S1 Dataset Result values of model fitting in the healthy group. (XLSX)

S2 Dataset Result values of direct characterization and model fitting in Fig 7. (XLSX)

S1 Supplementary Material Acquisition of ASL reactive hyperemia and result images. This supplementary file includes ASL sequence settings, image processing, quantification of perfusion, result perfusion maps, and comparison of perfusion-time curves between the healthy and patient group.
(PDF)

## Abbreviations

ABI: Ankle-brachial index
ASL: Arterial spin labeling
ATP: Adenosine triphosphate
CV: Coefficient of variation
PAD: Peripheral arterial disease
RBC: Red blood cell
ROI: Region of interest
TTP: Time-to-peak
