# Supplementary material for: A physiological model for interpretation of arterial spin labeling reactive hyperemia of calf muscles
Source: PLoS One. 2017 Aug 24;12(8):e0183259. doi: 10.1371/journal.pone.0183259 (PMC5570335; doi:10.1371/journal.pone.0183259)
Supplement: S1 Supplementary Material — This supplementary file includes ASL sequence settings, image processing, quantification of perfusion, result perfusion maps, and comparison of perfusion-time curves between the healthy and patient group. (PDF) [file pone.0183259.s004.pdf]

Supplementary material

The informed consent of the participants in the ASL perfusion study, including 7 healthy volunteers and 9 patients with PAD, was obtained following a protocol approved by the institutional Research Ethics Board. Both groups excluded individuals with vascular stents in the thigh and other MRI contraindication. Imaging was performed at 3 Tesla (MR 750 by GE, Milwaukee, Wisconsin) with an 8-channel cardiac receive array placed in the calf region. The subjects were in a foot-first supine position and rested for 5 minutes before imaging started. Flow interruption was achieved by thigh compression with a 12 cm long air cuff, which was inflated to 220-250 mmHg within 10 s. The cuff was deflated within 3 s by opening the air valve after ischemic durations were reached. Repeated flow interruption in the healthy group was separated by at least 15 minutes of rest. The images were reconstructed and processed in MATLAB (MathWorks, Natick, MA). Statistical analysis was performed in Prism (GraphPad Software, San Diego, CA).

The healthy group consisted of subjects younger than 30 years old without known cardiovascular disease. All 7 subjects underwent 2, 3, and 5 min of cuff-induced ischemia; five of them also participated in a 1-min ischemia experiment. The ischemic durations were applied in random order and separated into two sessions scanned on different days. The ASL scan was started 30 s before deflation of the air-cuff and continuing for at least 2 min. Baseline perfusion at rest was recorded for 2 min before any stress on the first day.

The median age of the patient group was 74.5 yrs (51 to 83). The ABI ranged from 0.3 to 0.87. Seven patients had claudication, and the remaining two had rest pain. The patients only underwent 2-min ischemia once for the more symptomatic leg. The ASL scan was started before flow interruption and lasted for 5.5 minutes, therefore including 1 minute of rest, 2 minutes of ischemia, and 2.5 minutes of reperfusion.

ASL settings

Reactive hyperemia was recorded with flow-sensitive alternating inversion recovery (FAIR, a version of pulsed ASL). The parameters of the FAIR sequence included single-slice steady-state free precession acquisition, with field of view = 16×16 cm^2, in-plane matrix size = 64×64, slice thickness = 1 cm, TE/TR = 1.7/3.8 ms, flip angle = 70°, and PLD = 1.4 s. The acquisition was repeated every 3.35 s. Q2TIPS (Quantitative Imaging of Perfusion Using a Single Subtraction II with thin-slice TI_1 periodic saturation) was applied to reduce the sensitivity to blood transit time, with the train of saturation pulses starting at TI_1=700 ms and ending 100 ms before image acquisition[1]. To suppress background signal, a saturation pulse was applied at the imaging plane prior to labeling, followed by two non-selective inversion pulses at 560 and 1200 ms after the saturation pulse.

Image processing and perfusion quantification

The region of interest (ROI) was manually drawn based on the reference images to cover the entire muscle region. The arterial voxels exhibited large temporal standard deviation of ASL images and were excluded from the ROI. In some cases, cuff inflation caused a vertical shift of more than one voxel, which was corrected by shifting the images during ischemia to align with the rest of the images. Cases with displacement smaller than one voxel were not corrected. The data points experiencing cuff inflation and deflation were excluded from quantification. To minimize the artifact of interleaved acquisition, both the label and control series were linearly interpolated, followed by subtraction between time-matched label and control data [2]. The difference signal was corrected for background suppression by multiplying the difference signal by $(1.06)^2$ [3]. The resulting difference signal ΔM was converted to perfusion f_{ASL} :

$$f_{ASL}(t) = \frac{\lambda}{2TI_1} \frac{\Delta M(t)}{M_{0b}} e^{-PLD/T_{1b}}$$

where M_{0B} is the signal of fully relaxed blood, which was very close to the baseline signal of muscle tissue acquired with SSFP before stress[4], and λ is the blood volume partition coefficient assumed to be 0.9 mL/g.

Results of ASL reactive hyperemia

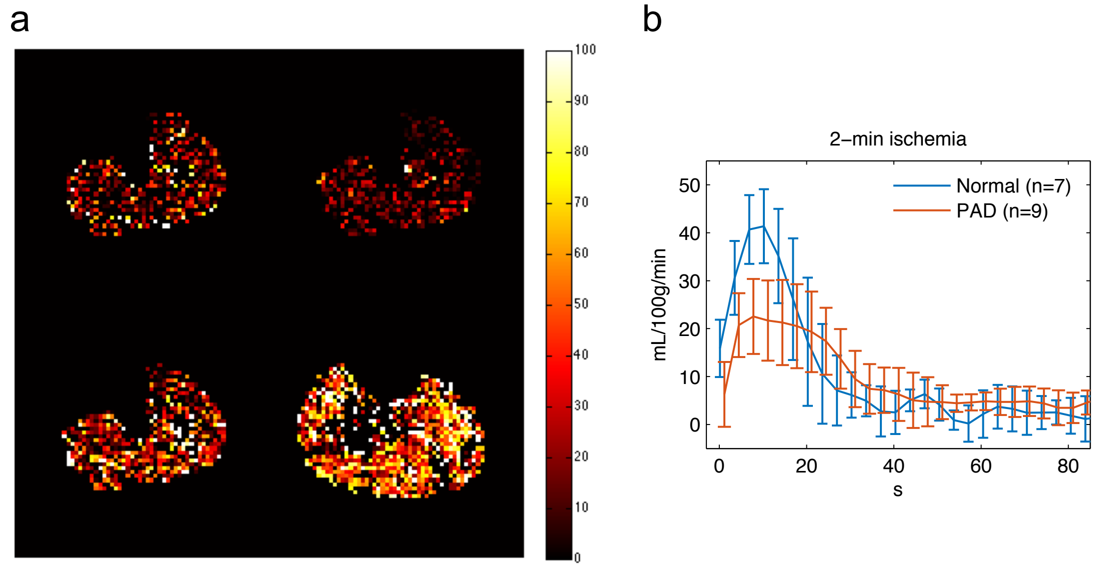

(a) Baseline, ischemia, and hyperemia perfusion maps of a patient (from top left, top right, and bottom left, respectively) and a hyperemia perfusion map of a healthy subject (bottom right) with 2-min ischemia. Each perfusion map represents an average of 5 consecutive difference images; hence the hyperemia maps represents the averaged perfusion of the first 33 s after ischemia. (b) The time course mean and standard deviation of reactive hyperemia in the healthy and patient group.

References

1. Luh WM, Wong EC, Bandettini PA, Hyde JS. QUIPSS II with thin-slice TI1 periodic saturation: a method for improving accuracy of quantitative perfusion imaging using pulsed arterial spin labeling. Magn Reson Med 1999;41:1246–1254.

2. Lu H, Donahue MJ, van Zijl PCM. Detrimental effects of BOLD signal in arterial spin labeling fMRI at high field strength. Magn Reson Med 2006;56:546–552.

3. Garcia DM, Duhamel G, Alsop DC. Efficiency of inversion pulses for background suppressed arterial spin labeling. Magn Reson Med 2005;54:366–372.

4. Zun Z, Wong EC, Nayak KS. Assessment of myocardial blood flow (MBF) in humans using arterial spin labeling (ASL): feasibility and noise analysis. Magn Reson Med 2009;62:975–983.
